# Supplementary material for: Retrospective methodology to estimate daily infections from deaths (REMEDID) in COVID-19: the Spain case study
Source: Sci Rep. 2021 May 28;11:11274. doi: 10.1038/s41598-021-90051-7 (PMC8163852; doi:10.1038/s41598-021-90051-7)
Supplement: Supplementary file 1 — Supplementary Information. [file 41598_2021_90051_MOESM1_ESM.docx]

Supplementary material. Validation test

Retrospective Methodology to Estimate Daily Infections from Deaths (REMEDID) in COVID-19: the Spain case study

David García-García^1^, María Isabel Vigo^1^, Eva S. Fonfría^2^, Zaida Herrador^3^, Miriam Navarro^2^, Cesar Bordehore ^2,4*^

^1^ Department of Applied Mathematics, University of Alicante, Alicante, Spain.

^2^ Multidisciplinary Institute for Environmental Studies “Ramon Margalef”, University of Alicante, Alicante, Spain.

^3^ National Centre of Epidemiology, Instituto de Salud Carlos III, Madrid, Spain.

^4^ Department of Ecology, University of Alicante, Alicante, Spain.

Corresponding author:

Dr. Cesar Bordehore

Multidisciplinary Institute for Environmental Studies “Ramon Margalef” & Department of Ecology, University of Alicante, Campus San Vicente del Raspeig, 03690, Spain

Tel: +34 5903400 ext 8755, Fax: +34 965903625

cesar.bordehore@ua.es

**Validation test**

In order to validate REMEDID, it would be desirable to have an accurate time series of daily infections and deaths. Then, the REMEDID infections inferred from deaths could be compared to the recorded infections. However, such datasets are not available because many infections in any disease or plague are usually undetected. On the other hand, REMEDID-estimated infections cannot be compared to those of dynamical models, which also need accurate data to be tuned. In fact, the main motivation for developing REMEDID has been to provide (more) reliable data to feed dynamical models.

As an alternative of validation, we propose the following experiment as a proof of concept. In the official COVID-19 time series, *IO_21_*, infections are given the date of symptoms onset. Then, REMEDID applied to official deaths with the PDF of illness onset to death, *h(t)*, instead of the PDF of infection to death, *f(t)*, should produce a time series comparable to *IO_21_*. Official accumulated cases and deaths on 22 June 2020 were 261,111 and 29,676, respectively, which gives a CFR of 11.37%. This CFR is overestimated due to an under detection of cases in that period. On the other hand, official accumulated cases and deaths from 23 June to 29 November 2020 were 1,482,293 and 17,439, which gives a CFR of 1.18%. Although closer to reality, this value is still overestimated. In any case, if we use these CFR values to construct the segmented timeseries of CFRs, as in Section 2.2, when applying REMEDID the resulting estimated daily infections should be comparable to *IO_21_*. The results are shown in Figure S.1 where the REMEDID estimated daily infections is in very good agreement with the smoothed *IO_21_* (14-day running mean), with a correlation between the two series of 0.98 % (p-value<0.001). Because smoothed time series present a high degree of serial correlation, the correlation and the significance level was estimated according to a specific Monte Carlo analysis based on the randomization of phases in the frequency domain^1^. Notice that (i) application of the 14-day running mean to the *IO_21_* series removes the weekly variability due to the weekend infections misreport; (ii) the agreement of the two series would improve with an improved estimate of the CFR time series. Thus, the good match between the estimated and the original daily infections timeseries proves the theoretical concept that supports REMEDID.

Note the differences with the results presented in section 3.2, where the PDF includes the incubation period and a more realistic CFR, as estimated from the National Seroprevalence Study data.

**References**

1. Ebisuzaki, W. A method to estimate the statistical significance of a correlation when the data are serially correlated, J. Clim., 10, 2147–2153, (1997).


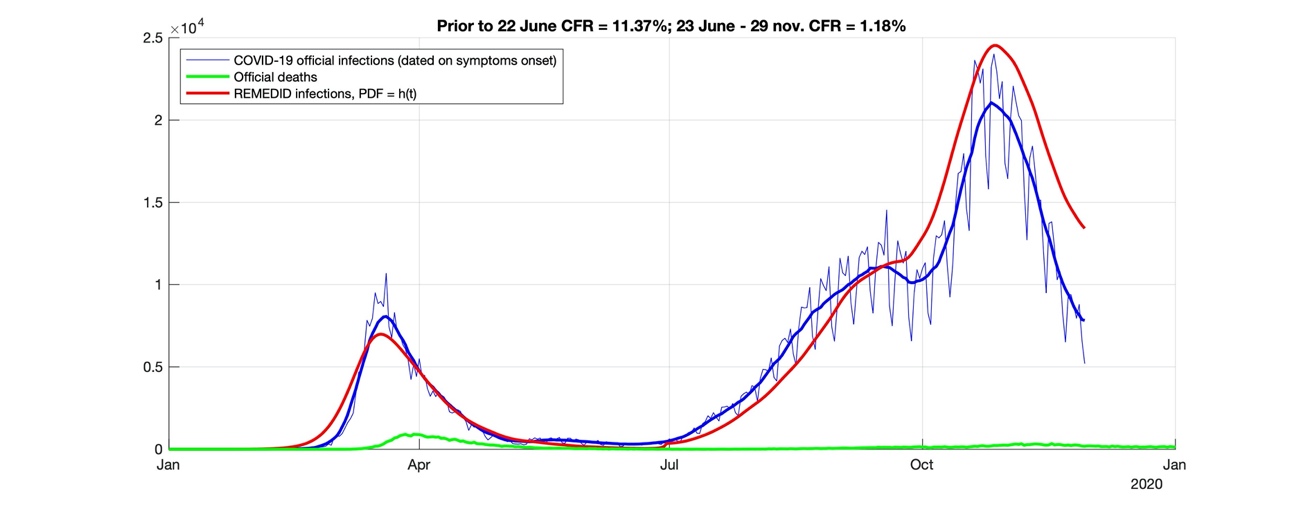


Figure S.1. REMEDID validation test. Thin blue line is official infections and thick blue line is its 14-day-running mean; green line is official deaths; and red line is REMEDID applied with the PDF of illness onset to death, *h(t)*.
